# Supplementary material for: The ‘what’ and ‘how’ of screening for social needs in healthcare settings: a scoping review
Source: PeerJ. 2023 Apr 21;11:e15263. doi: 10.7717/peerj.15263 (PMC10124546; doi:10.7717/peerj.15263)
Supplement: Supplemental Information 2 [file peerj-11-15263-s002.docx]

**Appendix 1**: Search Strategy

**Database:**
Ovid MEDLINE(R) ALL <1946 to April 29, 2022>

| **#** | **Query** |
| --- | --- |
| 1 | ((social determinant* or social risk* or social need* or social factor*) adj3 (screen* or identif* or evaluat* or assess*)).m_titl. |
| 2 | limit 1 to (humans and yr="2010 -Current") |
